# Overexpression of sorcin in multidrug-resistant human breast cancer

**DOI:** 10.3892/ol.2014.2543

**Published:** 2014-09-17

**Authors:** ZHAOHUA GONG, PING SUN, HONGJIN CHU, HUA ZHU, DENGJUN SUN, JIAN CHEN

**Affiliations:** 1 Department of Oncology, Yantai Yuhuangding Hospital Affiliated to Qingdao University, Yantai, Shandong 264000, P.R. China; 2 Central Laboratory, Yantai Yuhuangding Hospital Affiliated to Qingdao University, Yantai, Shandong 264000, P.R. China

**Keywords:** sorcin, neoadjuvant chemotherapy, breast cancer

## Abstract

Sorcin is a soluble resistance-related calcium-binding protein, which is expressed in normal mammalian tissues, such as the liver, lungs and heart. It has been observed to be elevated in a number of cancer types, including colorectal, gastric and breast cancer. Its upregulation is usually associated with the development of chemotherapeutic drug resistance. The aim of this study was to evaluate the sorcin expression levels in human serum samples of breast cancer subjects at various stages, and subsequently compare the outcome of neoadjuvant chemotherapy when the sorcin levels fluctuated. In total, 50 subjects were recruited from patients who were admitted to Yantai Yuhunagding Hospital (Yantai, China) and diagnosed with breast cancer. Blood samples prior to and following chemotherapy were assessed using two-dimensional gel electrophoresis (2-DE) and western blot analysis. The 2-DE analysis of the serum samples revealed that sorcin was upregulated in six out of 29 neoadjuvant chemotherapy (NAC)-sensitive patients and, in those who developed multidrug resistance, sorcin was upregulated in 15 out of 21 patients (P<0.01). The differential expression levels of sorcin were confirmed by western blot and immunohistochemical analysis. In conclusion, sorcin expression in the human serum of breast cancer patients who are resistant to NAC was elevated when compared with that of NAC-sensitive patients.

## Introduction

Sorcin, a soluble resistance-related calcium-binding protein, was initially observed to be overproduced in the vincristine-resistant DC-3F/VCR-5 Chinese hamster cell line, by Meyers and Biedler (1). Following this, it was also identified in drug-resistant mice and human cell lines (2–4). Sorcin is expressed in numerous tissues in mammals, such as the liver, lungs and, most abundantly, cardiac myocytes. Its expression in normal mammalian tissues is highly conserved.

Sorcin is a cytoplasmic protein that is tightly associated with free ribosomes, rough endoplasmic reticulum cisternae, mitochondria, nuclear membrane and microtubules (5). Biological characteristic studies of sorcin have confirmed a molecular mass of 22 kDa and determined that it is part of the penta-EF-hand (PEF) protein family, with typical calcium-binding sites located in the first pair of EF-hands (4,6,7).

The overexpression of sorcin has been reported in a number of tumor-resistant cell lines. Increasingly convincing evidence has suggested that sorcin is involved in survival mechanisms responsible for multidrug resistance and is associated with a poor prognosis during the therapeutic treatment of cancer patients (8). To date, the overexpression of sorcin has been observed in a number of multidrug-resistant (MDR) cell lines and several tumor cell types, including human colorectal cancer cells, human gastric cancer cells (9,10), leukemia (11,12), ovarian and breast cancer cells (13,14) and lung cancer (15). Our unpublished data, regarding the correlation between the expression levels of sorcin and the outcome of neoadjuvant chemotherapy (NAC) in breast cancer patients, showed that the remission rate was significantly higher in patients with low expression levels of sorcin than in patients with high sorcin expression levels, and that the expression of sorcin was reduced following treatment. It was hypothesized that the expression level of sorcin in breast cancer may predict the efficiency of the paclitaxel/epirubicin regimen in NAC.

To date, laboratory detections of sorcin expression are predominantly at the mRNA and protein level using methods of proteomics, including reverse transcription-polymerase chain reaction, western blot analysis and immunohistochemistry (IHC), subsequent to siRNA transfection. At the DNA level, sorcin expression has also been tested using microarray and northern blot analysis (12,13). Two-dimensional gel electrophoresis (2-DE) is one of the most commonly used techniques in proteomics. It is widely used to study protein expression patterns in a variety of cell lines (11). Over the last two decades, this approach has been used for profiling expression patterns in cancer and in cancer cells with multidrug resistance, which enables the identification of proteins that are involved in tumorigenesis and multidrug resistance for specific drugs. Therefore, methods of proteomics and immunohistochemistry were investigated in this study to assess the role of sorcin in a phenotype of breast cancer with multidrug resistance.

Understanding this protein may provide targeted therapeutic applications among cancer patients. Sorcin may be a potential prognostic marker for a number of malignancies, including acute leukemia and breast cancer, which is of particular relevance in the current study. However, the mechanisms whereby sorcin is interrelated with multidrug resistance may vary in different cancer cells (16).

## Materials and methods

### Sample preparation

Serum samples were extracted from 30 stage III and IV breast cancer patients who received preoperative NAC and were recruited for a prospective preoperative clinical trial at the Yantai Yuhuangding Hospital (Yantai, China) between 2008 and 2010. The patients suffered from locally advanced breast cancer, in which the purpose of neoadjuvant treatment was to downstage the cancer for an improved chance of complete resection, or high operative risks were anticipated due to old age or comorbidities, which prevented them from undergoing initial surgical treatment. Among the subjects, 20 patients received two cycles of 175 mg/m^2^ paclitaxel and 80 mg/m^2^ epirubicin on day one, once every three weeks, while 10 patients received two cycles of 75 mg/m^2^ docetaxel and 80 mg/m^2^ epirubicin on day one, once every three weeks. In total, 24 patients responded to the chemotherapy [complete response (CR), partial response (PR) and stable disease (SD)] and 18 patients developed progressive disease (PD). All samples were removed with patient consent.

Blood samples were drawn prior to each chemotherapy course, including a baseline pretreatment sample (day zero) and a post-chemotherapy (week six), but prior to the third course of chemotherapy or surgery, sample. Blood samples were subsequently centrifuged at 1,200 × g for 20 min at 4°C, and the supernatant was aliquoted and stored at −80°C until use. This biomarker study was approved by the institutional review board at Yantai Yuhuangding Hospital and a waiver of informed consent was granted.

### Serum preparation (extraction of serum from human subject whole blood)

Serum samples were prepared according to the manufacturer’s instructions in the Proteoprep kit, Sigma-Aldrich (St. Louis, MO, USA). In total, 300 μl of the protein extraction reagent, mixed with high purity water, was added to 400 μl of the equilibration buffer, and then subjected to centrifugation at 5,000 × g for 5–10 sec at −4°C and repeated once. Following this, 25–50 μl of each serum sample was diluted to 100 μl with the equilibration buffer and mixed thoroughly. This sample was then added to the top of the column and incubated for 5–10 min at room temperature, and subsequently centrifuged at 8,000 × g for 60 sec at −4°C and repeated once. In the final step, 125 μl of equilibration buffer was added prior to storage of the samples at −20°C.

### Extraction of salt with acetone and protein solubilization

Serum samples were initially precipitated with acetone. Cold acetone (Fluka Biochemika, Buchs, Switzerland) was added to a serum sample at a ratio of 1:4 and stored at −20°C for 2 h, following which, centrifugation was conducted at 12,000 × g for 30 min. The supernatant was discarded and the pellet was dissolved in 200 μl of the lysis buffer containing 40 mM Tris, 7 M urea, 2 M thiourea, 2% 3-[(3-cholamidopropyl) dimethylammonio]-1propanesulfonate (CHAPS), 65 mM dithiothreitol (DTT) and 1% IPG Buffer (GE Healthcare, Little Chalfont, UK).

### 2-DE analysis

Protein concentration was determined using the 2D-Quant Kit (Amersham Pharmacia Biotech, Amersham, UK), according to the manufacturer’s instructions. In total, 500 μg of each pooled protein sample was diluted in the rehydration buffer (7 M urea, 18 mM DTT, 4% CHAPS, 0.5% IPG buffer and 0.002% bromophenol blue). The isoelectric focusing was performed on the Ettan IPGphor II (GE Healthcare Bio-Sciences, Piscataway, NJ, USA). The IPG strips were initially rehydrated at 30 V for 12 h, and subsequently focused at 500 V for 1 h, 1,000 V for 1 h, 3,000 V for 3 h and 5,000 V for 3 h, and then maintained at 8,000 V until a total of 50,000 V/hr was achieved. Following isoelectric focusing, the IPG strips were equilibrated with 1.5 M Tris-HCl (pH 8.8), 6 M urea, 87% glycerol, 2% sodium dodecyl sulfate (SDS) and 0.2% bromophenol blue. The IPG strips were initially treated with 15 ml 1% DTT for 10 min with constant shaking, followed by alkylation with 15 ml 2.5% indole-3-acetic acid for 15 min.

The equilibrated strips were transferred to 12.5% SDS polyacrylamide gel electrophoresis (SDS-PAGE) on the Ettan DALT twelve system (GE Healthcare) with constant power (0.2 W/gel, 1 h; 0.4 W/gel, 1 h; 250V, 4.5 h). The gels were stained with Coomassie blue R350 (GE Healthcare), and scanned using a PowerLook 2100 XL scanner system (Umax Technologies, Taipei, Taiwan).

Spots of interest were excised from gels stained by Coomassie Blue R350, and were digested with sequencing grade modified trypsin (Promega Corporation, Madison, WI, USA) using the following protocol: The colloidal particle was soaked in decoloring working liquid (50% acetonitrile/50 mmol/l NH_4_HCO_3_) in 37°C water bath until the blue faded away. The decolored blue dots were dehydrated for 15 min with 100% acetonitrile, and subsequently spun briefly. Trypsin solution was added to the dried colloidal particle (10 μg/μl trypsin, 100 mmol/l NH_4_HCO_3_ and 125 mmol/l CaCl_2_) at 4°C and bulged for 30 min. The excessive trypsin epispastics were removed and 10 μl 25 mmol/l NH_4_HCO_3_ enzyme solution was added and the resulting mixture was incubated at 37°C overnight. Following the completion of the enzyme solution preparation, the extract liquor [acetonitrile (ACN) 50% + 5% trifluoroacetic acid (TFA)] was used to extract the peptides and the solution was heated at 37°C for 1 h. The liquid supernatant was extracted to a new microcentrifuge tube, enriched and dried by centrifugation. The dissolved and dried peptides were subsequently used with 3 μl diluent (ACN 30% +1 % TFA) for mass spectrometry detection.

### Mass spectrum identification

Subsequent protein identification was conducted on the ABI 4700 Proteomic Analyzer MALDI-TOF-MS/MS mass spectrometer (Life Technologies, Carlsbad, CA, USA) in the reflective mode. The peptide mass fingerprint (PMF) was acquired between 800–3,500 Da. In total, 24 peaks from the PMF were selected to obtain the MS/MS spectra. The PMF and MS/MS results were then searched against a human subset of the Swiss-Prot database using the GPS explorer software (Life Technologies).

### Western blot analysis

In total, 100 μg serum sample was diluted with an equal amount of loading buffer (12% SDS; 135 mM Tris-HCl, pH 6.8; 20% glycerol; 0.02% bromophenol blue and 10% 3-mercaptoethanol). The samples were subjected to polyacrylamide gel electrophoresis and transferred onto a polyvinylidene difluoride membrane (Millipore, Massachusetts, CA, USA). The membranes were treated with Tris-buffered saline with 0.1% Tween-20 containing 5% dried non-fat milk at 4°C overnight. The membranes were then immunoblotted with the monoclonal mouse anti-human sorcin antibody (sc-100859; Santa Cruz Biotechnology, Inc., Santa Cruz, CA, USA) and the monoclonal rabbit anti-avian β-actin antibody) sc-47778; Santa Cruz Biotechnology, Inc.) at 4°C overnight. The following day, the membranes were washed three times with phosphate-buffered saline containing 0.05% Tween-20 and incubated with a polyclonal goat anti-mouse peroxidase-conjugated secondary antibody (ZSGB-BIO, Beijing, China) at room temperature for 1 h. The immunoreactive blots were identified using the chemiluminescence detection ECL plus kit (GE healthcare, Buckinghamshire, UK) and the LAS 3000 Lumino-image analyzer (Fujifilm, Tokyo, Japan). The band intensity was analyzed by using the NIH image analysis software (NIH, Bethesda, ML, USA).

### Statistical analysis

The χ^2^ test was used to establish the statistical significance between the expression levels of sorcin and the number of patients who developed resistance to NAC. P<0.01 was considered to indicate a statistically significant difference (Table I).

## Results

### Sorcin upregulation in MDR patients

According to the Response Evaluation Criteria in Solid Tumors (17), the subjects were grouped into CR, PR, SD and PD. In the current study, patients that were evaulated as CR, PR and SD were predicted to be responsive to NAC treatment. Therefore, among the 50 breast cancer patients included in this study, 29 responded well to the NAC (58%), whereas 21 patients developed multidrug resistance (42%) (Table I). The 2-DE analysis of the serum samples revealed that sorcin was upregulated in six out of 29 (20.7%) NAC-sensitive patients and, in those who developed multidrug resistance, sorcin was upregulated in 15 out of 21 patients (71.4%).

**Figure 1:**
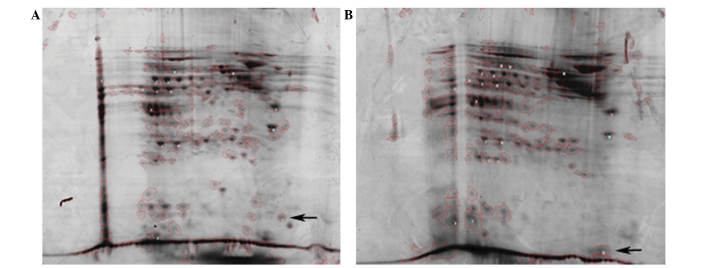
Two-dimensional gel electrophoresis maps of serum obtained form breast cancer patients who (A) were neoadjuvant chemotherapy-sensitive or (B) developed multiple drug resistance. The protein spots representing sorcin are highlighted by arrows.

The sample pools of NAC-sensitive and MDR patients were run separately using 2-DE analysis; a protein spot of sorcin was identified on the 2-DE map loaded with serum samples from the NAC-resistant patients (Fig. 1B) as well as a further 19 distinguishable protein spots (results not shown). Each pool was analyzed three times.

### Western blot analysis of MDR patients

**Figure 2:**
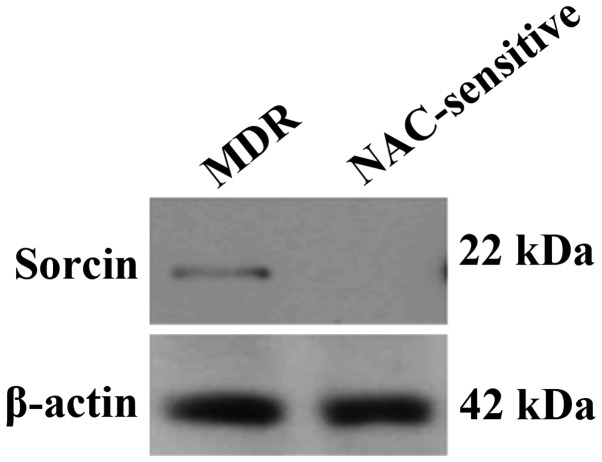
Western blot analysis of sorcin and β-actin expression in sample serum from breast cancer patients who were NAC-sensitive or who developed multidrug resistance, versus normal subjects. NAC, neoadjuvant chemotherapy; MDR, multidrug resistant.

Western blot analysis using anti-sorcin antibodies revealed a specific band in the sample pool of MDR serum, whereas no band was visible for the NAC-sensitive group (Fig. 2). Anti-β-actin antibodies were used as a control for the analysis.

### IHC

**Figure 3:**
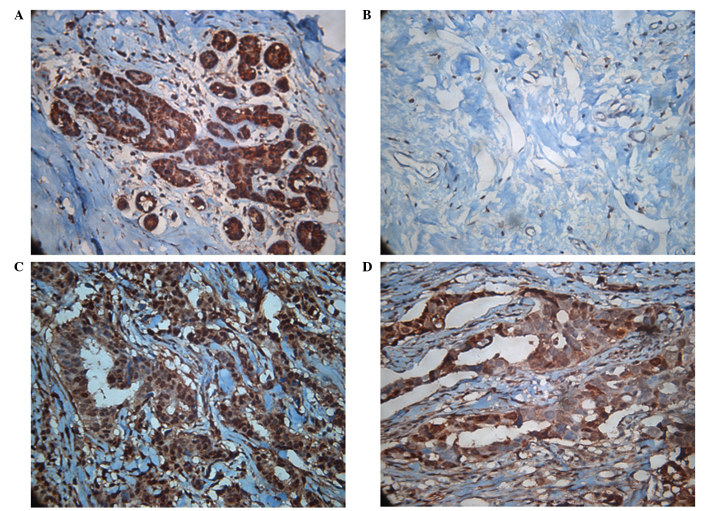
Validation of sorcin expression of infiltrating ductal breast cancer tissues by immunohistochemical staining. (A) Representative biopsy taken from patients who responded effectively to neoadjuvant chemotherapy, showing low sorcin expression. (B) Biopsy of the same patient following chemotherapy. (C) Representative biopsy taken from patients who did not respond to neoadjuavnt chemoptherapy, prior to chemotherapy. (D) Biopsy of the same patient following chemotherapy. Staining is evident in the cytoplasm, which is represented on the figure with dark brown color. Magnification ×200.

Furthermore, as predicted, IHC of the infiltrating ductal breast cancerous tissue revealed heavy staining of sorcin in tissues obtained from patients with developed resistance to NAC. The results from two cases are shown in Fig. 3, where staining is particularly evident in the cytoplasm.

## Discussion

This study provides evidence of the involvement of sorcin in the development of drug resistance in breast cancer. Using 2-DE and western blot analysis, sorcin was identified in the blood serum of human breast cancer subjects and new insights into the manner by which the expression levels of sorcin affect the outcome of NAC have been presented. The 2-DE analysis of the pooled sample serum of breast cancer patients revealed the upregulation of sorcin in >70% of all participants who did not respond to NAC (those with PD). Subsequent western blot analysis confirmed a positive band for patients who developed multidrug resistance, in contrast to those who were responsive to NAC. Furthermore, IHC staining of the cancerous tissue biopsy confirmed the sorcin upregulation. To the best of our knowledge, this is one of few studies that uses human serum as the test sample, as the majority of studies investigating the sorcin expression in tumors use tissues or cell lines. IHC staining of the breast tissue of patients from biopsies prior to and following NAC indicate that patients who responded well to NAC have significantly reduced sorcin expression in the cytosol, following NAC.

The majority of studies investigating sorcin expression have employed cell lines that are commercially available and engineered using cDNA cloning or siRNA (14,18). Additionally, by examining the resistance of particular cancer cells in apoptosis, the mechanism of sorcin in the prognosis of cancer maybe disclosed (11). Proteomic studies of organelle compartmentalization of sorcin have also been thoroughly investigated, predominantly through direct cell fractionation or following the treatment of target cell lines with antiblastic agents, such gemcitabine (15) and 5-fluorouracil (19). The current study, however, utilized serum samples acquired directly from breast cancer patients, and compared the sorcin levels between patients who presented with a resistant phenotype for chemotherapy and those who were sensitive to NAC. To the best of our knowledge, studies analyzing sorcin levels using human serum are limited. To date, the mechanisms of the occurrence, development, metastasis or resistance of breast cancer are not fully understood. Research is ongoing to locate suitable markers at the molecular level; sorcin is one of the numerous protein markers that is being systematically studied (20), and we hypothesize that the role of sorcin in predicting the chemotherapeutic response and aggressiveness of the malignancy is closely associated with its causative effect in multidrug resistance.

Breast cancer is one of the leading causes of cancer-related mortality in females worldwide (21); it is associated with high morbidity, poor prognosis and high metastatic rates (22). Predominantly, breast cancer cell resistance to antiblastic cells is most likely a causative factor in therapeutic failure (23). As sorcin is considered a pivotal breast cancer resistance-related protein, understanding the mechanisms of sorcin at a molecular level may have a significant impact on the clinical management of breast carcinoma. IHC has been used to study the expression of sorcin in breast cancer tissue (24). Liu *et al* (25) demonstrated that 85.1% (40/47) of postoperative samples from breast cancer patients positively express sorcin. This may be partially associated with the presence of the progesterone receptor, overall survival rate and disease-free survival, but is not likely to be associated with the prognosis or clinical manifestation.

However, another study demonstrated that sorcin was only involved in the development of low-level paclitaxel resistance when full-length sorcin cDNA was transfected into MCF-7 human breast cancer cells, which are estrogen receptor-positive, and MDA-MB435S (parental MCF-7) cells (14). However, the overexpression of P-glycoprotein (P-gp) did not correlate with the degree of resistance in the paclitaxel-resistant human ovarian carcinoma subline, MCF-7. Therefore, it was speculated that sorcin may cause paclitaxel resistance in breast cancer, and may be dependent on the presence of estrogen receptors. Additionally, Kawakami *et al* (16) demonstrated that if sorcin was knocked down from an MDR1/P-gp-overexpressing MDR subline established from the human cervical carcinoma cell line, HeLa, the level of MDR1, which modulates the MDR1/P-gp transporter, was increased. Together with the increased level of caspase-3, it was hypothesized that the downregulation of sorcin may elevate the intracellular levels of calcium via the upregulation of MDR1 and thus, activated caspase-3 may induce apoptosis. Furthermore, by examining 25 breast cancer patient samples for P-gp expression, Zhao *et al* (26) demonstrated that minimal MDR1 mRNA expression may also lead to a MDR phenotype. P-gp is not expressed in normal breast tissue; however, it may be observed in cancerous breast tissue and normal peritumoral tissue, a common phenomenon that can be applied to the majority of malignancies (27).

To conclude, the upregulation of sorcin in the serum of breast cancer patients may be partially responsible for the development of multidrug resistance. NAC moderately reduces sorcin expression; however this does not occur in all breast cancer cases. The mechanism by which sorcin affects the development of multidrug resistance and patient response to NAC remains unknown. Although sorcin may be a potential prognostic marker for predicting the treatment outcome in breast cancer patients and possibly further malignancies, the mechanism of sorcin and the association with multidrug resistance may differ across cancer cell types. Gaining an improved understanding of this protein may provide targeted therapeutic applications among cancer patients.

## Figures and Tables

**Table I:** Patient responses, exhibiting an up- or downregulation in sorcin levels among patients who were NAC-sensitive or -resistant, respectively.

Subgroups	Total, n	Upregulated sorcin, n	Downregulated sorcin, n	χ^2^	P-value*
NAC-sensitive	29	6	23	12.87	<0.01
MDR	21	15	6		

* Calculated using the χ^2^ test.

NAC, neoadjuvant chemotherapy; MDR, multiple drug resistance.
